# The Non-Coding RNA Journal Club: Highlights on Recent Papers—4

**DOI:** 10.3390/ncrna2030009

**Published:** 2016-09-21

**Authors:** Daniel Gautheret, Joseph H. Taube, Sendurai A. Mani, Gaetano Santulli, Diego Cuerda-Gil, R. Keith Slotkin, Bo Zhang, Yanli Wang, David W. Salzman, Joanne B. Weidhaas

**Affiliations:** 1 Institute for Integrative Biology of the Cell CNRS, CEA, Universite Paris-Sud. 91198 Gif sur Yvette, France; daniel.gautheret@u-psud.fr; 2 Department of Biology, Baylor University, Waco, TX 76798, USA; Joseph_Taube@baylor.edu; 3 Department of Translational Molecular Pathology, The University of Texas MD Anderson Cancer Center, Houston, TX 77030, USA; smani@mdanderson.org; 4 Metastasis Research Center, The University of Texas MD Anderson Cancer Center, Houston, TX 77030, USA; 5 College of Physicians & Surgeons, Columbia University Medical Center, 1150 St Nicholas Avenue, New York, NY 10032, USA; gsantulli001@gmail.com; 6 Department of Molecular Genetics, Ohio State University, 570 Aronoff Laboratory, 318 West 12th Avenue, Columbus, OH 43210, USA; cuerdagil.1@osu.edu (D.C.-G.); slotkin.2@osu.edu (R.K.S); 7 Key Laboratory of RNA Biology, Institute of Biophysics, Chinese Academy of Sciences, Beijing 100101, China; bo.zhang.1@ulaval.ca (B.Z.); ylwang@ibp.ac.cn (Y.W.); 8 Department of Radiation Oncology, UCLA David Geffen School of Medicine, Professor, Director, Translational Research, Los Angeles, California 90024, USA; DSalzman@mednet.ucla.edu (D.W.S.); JWeidhaas@mednet.ucla.edu (J.B.W.)

## 1. Introduction

We are glad to share with you our **fourth** *Journal Club* and highlight some of the most interesting papers published recently. We hope to help keep you aware of non-coding RNA research outside of your area. The *Non-Coding RNA* Scientific Board wishes you an exciting and fruitful read. 

## 2. Disruptive RNA-Seq Bioinformatics

### Highlight by Daniel Gautheret

Quantifying gene expression from RNA-Sequencing (RNA-Seq) data used to be a settled matter. Nearly all protocols would start with “Step 1: align sequence reads onto a reference genome”. This process required days of computer time and access to powerful servers, even for a basic experimental setup. As RNA-Seq projects grew larger in scale, running this analysis started to turn into a bioinformatician's nightmare. This is now changing radically thanks to efforts by two US-based groups. Their new programs, Kallisto [1] and Salmon [2], are based on hashing, a method that converts a sequence file into an index of words of fixed size (k-mers). Comparing an RNA-Seq library to an entire transcriptome at the k-mer level now takes minutes instead of hours. Both programs implement procedures to resolve ambiguities (k-mers matching several transcripts) and assign a read count to each transcript, achieving a precision in transcript abundance estimation comparable to that of the best mapping-based methods. Not only are Salmon and Kallisto as accurate and an order of magnitude faster than the current software, but they are also more flexible, as they enable the quantification of independent transcripts as well as whole gene expression. The non-coding RNA community should take note.

## 3. Cytoplasmic lncRNA Mediates HIF-Targeted Enzymatic Cascade

### Highlight by Joseph H. Taube and Sendurai A. Mani

The potential functions of long non-coding RNAs, which were first established as nuclear regulators of gene expression, are expanding to include cytoplasmic roles. In an elegant blend of biochemistry, cell biology, and cancer biology, Lin, et al. [3] show that LINK-A (long intergenic non-coding RNA for kinase activation) functions in the cytoplasm by binding and altering the conformation of protein tyrosine kinase 6 (also known as breast tumor kinase, BRK). By recruiting a second kinase, Leucine-rich repeat kinase 2 (LRRK2), and by interacting with BRK at two separate domains to alter its conformation, LINK-A facilitates the phosphorylation and nuclear translocation of Hypoxia-inducible factor 1-alpha (HIF-1α), despite normoxic conditions. Notably, the expression of LINK-A and the activation of HIF-1α signaling is not only enriched in triple-negative breast cancer but is also predictive of poor recurrence-free survival in triple-negative breast cancer (TNBC) patients. Importantly, blocking LINK-A via shRNA compromises tumor growth, thus opening the door for lncRNA-targeted therapy. 

## 4. Microbiome and microRNAs Mediate the Communication Between Gut and Vasculature

### Highlight by Gaetano Santulli

The gut microbiome has been implicated in the pathogenesis of endothelial dysfunction and atherosclerosis. Ajit Vikram and colleagues have elegantly identified one of the mechanisms underlying such a connection in microRNAs (miRs) [4]. In particular, the authors found that the expression of miR-204 in the vascular wall is remotely upregulated by the microbiome in mice. One of the targets of miR-204 is Sirtuin1 (Sirt1), a class II histone deacetylase that stimulates endothelial nitric oxide synthase (eNOS), which is essential for the maintenance of a normal vascular tone [5]. Intriguingly, a high-fat diet upregulated miR-204 in the aorta and the suppression of gut flora biomass resulted in a significant decrease in vascular miR-204 and an increase in the expression of Sirt1, with a general amelioration of endothelial function.

This discovery has major implications given the acknowledged importance of diet in the pathogenesis of cardiovascular disease [6]. Moreover, the authors have left the door open for the identification of the mediators responsible for the remote communication between the gut and vessels, suggesting a potential role for serum factors. 

## 5. Stable Intronic Sequence RNAs (sisRNAs) Regulate Their Cognate Pre-mRNAs

### Highlight by Diego Cuerda-Gil and R. Keith Slotkin

Typical intron RNAs are rapidly degraded after they are spliced from their pre-mRNA. A new class of “stable intronic sequence RNAs (sisRNAs)” has been recently identified, that do not degrade and rather accumulate in the nucleus. SisRNAs are produced from human cell lines, introns of the Epstein-Barr virus, and Xenopus tropicalis oocytes, but they have poorly understood functions. Pek et al. [7] identified 34 candidate sisRNAs from *Drosophila melanogaster* embryos. They focused on one sisRNA, sisR-1, produced from the regena (rga) locus. Their data suggests that sisR-1 regulates a negative feedback loop influencing protein production from this locus. This function is mediated through the repression of a natural antisense transcript (called ASTR) produced from the rga locus. The authors propose that sisR-1 plays a critical role in the nucleus by repressing ASTR accumulation during embryogenesis, and therefore increases the robustness of rga protein production. Deeper characterization and understanding of sisRNAs are needed, including the determination of whether sisRNAs are evolutionary conserved. 

## 6. Structural Study of CRISPR-Cpf1 Still on the Road

### Highlight by Bo Zhang and Yanli Wang

Cpf1 is a newly identified single RNA-guide endonuclease of a class 2 type V CRISPR-Cas system. It has been successfully utilized to manipulate the human genome [8,9,10]. Recently, two kinds of crystal structures of Cpf1 have been reported [11,12,13]. In the LbCpf1-crRNA binary complex, the overall structure has a triangle-shape and a bilobed architecture. The repeat region of the crRNA forms a pseudoknot structure recognized by the WED and RuvC domains of Cpf1, whereas the spacer region is invisible in the binary complex. In the AsCfp1-crRNA-DNA ternary complex, the RNA-DNA heteroduplex is bound within the positively charged central channel, formed by the REC lobe and the NUC lobe. Structural comparison revealed that the REC lobe is significantly rearranged to accommodate the RNA-DNA heteroduplex, and that the PAM-interacting cleft undergoes a modest open-to-closed transition upon the PAM duplex binding. However, the cleavage mechanism of Cpf1 mediated target DNA cleavage still needs further studies. 

## 7. Ionizing Radiation Gets miRNAs Excited

### Highlight by David W. Salzman and Joanne B. Weidhaas

MicroRNA (miRNA) expression is regulated by several mechanisms, including transcription and processing, to generate a mature miRNA that is loaded into miRISC, which exacts gene silencing. It is widely accepted that miRNA expression is directly correlated with its gene silencing activity. However, in a recent Nature Communications article, Salzman et al. discovered that there is a pool of mature miR-34 in cells devoid of activity, lacking a canonical 5′-end phosphate, and is not loaded into miRISC. After DNA damage, this miR-34 is 5′-end phosphorylated through the DNA damage sensor ATM and the RNA kinase Clp1, thereby loading miR-34 into miRISC and activating it. Knockdown experiments differentiated two distinct miR-34 activation pathways following DNA damage. One pathway occurs via ATM/Clp1 activation and the other by TP53-mediated transcription. The ATM/Clp1-dependent pathway rapidly suppressed miR-34 target genes (CDK4 and BCL2) several hours ahead of TP53 transcription, which sustains miR-34 activity over time.

These findings highlight a discordance in miRNA expression and activity, and elucidate a novel mechanism for the activation of miRNA activity in response to an external stimulus. Numerous reports show that miRNA expression can be altered by external stimuli (environmental changes, growth factors, cell stress, drug treatment, etc.), and these findings raise questions about what the pre-stimulus pool of miRNAs in these systems are doing. Furthermore, this work supports the potential importance of miR-34 targeting to enhance radiation therapy in cancer patients [14].
