# Erratum: The Non-Coding RNA Journal Club: Highlights on Recent Papers—4. *Non-Coding RNA* 2016, *2*, 9

**DOI:** 10.3390/ncrna2040010

**Published:** 2016-09-30

**Authors:** Daniel Gautheret, Joseph H. Taube, Sendurai A. Mani, Gaetano Santulli, Diego Cuerda-Gil, R. Keith Slotkin, Bo Zhang, Yanli Wang, David W. Salzman, Joanne B. Weidhaas

**Affiliations:** 1 Institute for Integrative Biology of the Cell CNRS, CEA, Universite Paris-Sud. 91198 Gif sur Yvette, France; daniel.gautheret@u-psud.fr; 2 Department of Biology, Baylor University, Waco, Texas, USA; Joseph_Taube@baylor.edu; 3 Department of Translational Molecular Pathology, The University of Texas MD Anderson Cancer Center, Houston, Texas, USA; 4 Metastasis Research Center, The University of Texas MD Anderson Cancer Center, Houston, Texas, USA; smani@mdanderson.org; 5 College of Physicians & Surgeons, Columbia University Medical Center, 1150 St Nicholas Avenue, New York, NY 10032, USA; gsantulli001@gmail.com; 6 Department of Molecular Genetics, Ohio State University, 570 Aronoff Laboratory, 318 West 12th Avenue, Columbus, OH 43210, USA; cuerdagil.1@osu.edu (D.C.-G.); slotkin.2@osu.edu (R.K.S); 7 Key Laboratory of RNA Biology, Institute of Biophysics, Chinese Academy of Sciences, Beijing 100101, China; bo.zhang.1@ulaval.ca (B.Z.); ylwang@ibp.ac.cn (Y.W.); 8 Department of Radiation Oncology, UCLA David Geffen School of Medicine, Professor, Director, Translational Research, Los Angeles, California 90024, USA; DSalzman@mednet.ucla.edu (D.W.S); JWeidhaas@mednet.ucla.edu(J.B.W)

Please note that in the published editorial [1], affiliations 1, and 8 contained errors. The correct versions are as follows:

^1^ Institute for Integrative Biology of the Cell CNRS, CEA, Universite Paris-Sud. 91198 Gif sur Yvette, France.

^8^ Department of Radiation Oncology, UCLA David Geffen School of Medicine, Professor, Director, Translational Research, Los Angeles, California 90024, USA.

We apologize to readers for any inconvenience caused by these changes.
